# A Comprehensive Overview of Respiratory Compliance in Dogs Under General Anesthesia: Clinical Factors and Future Perspectives

**DOI:** 10.3390/ani15050746

**Published:** 2025-03-05

**Authors:** Tingfeng Xu, Ahmed S. Mandour, Kazumi Shimada, Lina Hamabe, Ryou Tanaka

**Affiliations:** 1 Veterinary Teaching Hospital, Tokyo University of Agriculture and Technology, Tokyo 183-8538, Japan; s243507x@st.go.tuat.ac.jp; 2 Department of Animal Medicine, Suez Canal University, Ismaila 41522, Egypt; dr_mandour@vet.suez.edu.eg; 3 Laboratory of Veterinary Surgery, Department of Veterinary Medicine, Tokyo University of Agriculture and Technology, Fuchu 183-8509, Japan; 4 Veterinary Clinical Oncology, Tokyo University of Agriculture and Technology, Tokyo 183-8538, Japan; linahamabe@go.tuat.ac.jp

**Keywords:** dogs, respiratory compliance, anesthesia monitoring, mechanical ventilation, thoracic surgery, laparoscopic surgery, abdominal surgery

## Abstract

Respiratory compliance reflects the ability of the lungs and chest wall to expand in response to increases in pressure. In this review, relevant studies were selected through a comprehensive literature search with the aim of summarizing and generalizing them to describe the relevant factors that may be present in veterinary clinical practice and affect respiratory compliance in dogs. Individual factors, including breeds, disease background, drugs administered, and especially surgical procedures, can result in alterations to respiratory compliance due to their impact on the respiratory system in dogs. Despite its potential clinical utility, such as in anesthesia monitoring, respiratory compliance remains underutilized in veterinary medicine, and further research is necessary to support its future clinical applications.

## Simple Summary

Respiratory compliance, encompassing pulmonary and chest wall compliance, is a key physiological parameter for assessing ventilation status, particularly in dog surgery and anesthesia monitoring. This review summarizes several important clinical factors influencing respiratory compliance and explores unresolved research questions in this field. The aim of this review is to provide a reference for future studies and provide evidence-based recommendations for anesthetists to optimize intraoperative respiratory compliance management to reduce unexpected anesthesia risk and avoid respiratory complications.

## 1. Introduction

Respiratory compliance consists of lung compliance and chest wall compliance. Lung compliance refers to the ability of the lungs to expand in response to pressure changes during breathing, while chest wall compliance measures the flexibility of the chest wall in accommodating these changes. However, since lung compliance and chest wall compliance are often difficult to consider separately, respiratory compliance is more commonly used to mean them together. Based on the methods of measurement, respiratory compliance can be categorized as either static (C_st_) or dynamic (C_dyn_) [1,2,3,4,5]. Specifically, respiratory compliance is defined as the change in lung volume (_Δ_V) per unit change in transmural pressure (_Δ_P) and is often measured clinically in milliliters per centimeter of water column (ml/cm H_2_O) or liters per centimeter of water column (L/cm H_2_O). In the field of veterinary medicine, C_st_ is utilized to assess the elasticity of the lungs and the compliance of lung tissue, facilitating the diagnosis of respiratory diseases. Conversely, C_dyn_ is employed to evaluate the ventilation status of the airways and the effectiveness of ventilation patterns during surgery [1,2,3,4,5,6,7,8,9] (Table 1).

The utilization of respiratory compliance is limited in veterinary medicine. Nevertheless, with the increasing utilization of thoracoscopic and laparoscopic surgery, employing respiratory compliance to evaluate lung tissue function and ventilation during surgical procedures has garnered interest. Respiratory compliance can be utilized as a method of evaluating the ventilation status of lung tissue to reduce the risk of lung tissue injury during laparoscopic surgery due to hyperventilation during alveolar recruitment maneuvers (ARMs) for lung protective ventilation (LPV) or as a result of increased intra-abdominal pressure (IAP) [10,11].

Spirometry is a conventional method employed to assess respiratory compliance; however, its utilization in veterinary clinics appears to be limited. This is primarily due to the challenges veterinarians face in maintaining prolonged control over a dog’s respiratory movements while it is awake and in the presence of a mask. The practicality of wearing such a mask for extended periods is particularly problematic in conscious dogs [12,13,14,15]. Consequently, ventilation monitoring modules such as the D-Lite/Pedi-Lite flow sensor from Datex are more commonly utilized in contemporary veterinary clinical practice. This module is based on Pitot tube values and employs computational methods to calculate respiratory compliance [12].

## 2. Purpose

The objective of this study is to examine the factors influencing dog respiratory compliance, with a specific focus on potential applications in anesthetic monitoring within a veterinary practice setting. This study will investigate the impact of factors such as dog breed, size, and body position on respiratory compliance, and will discuss the practical applications of respiratory compliance as an anesthesia monitoring tool in surgical procedures, with the aim of optimizing anesthesia management and reducing the risk of respiratory complications. Additionally, this study will provide a theoretical foundation for the future application of respiratory compliance as an anesthetic monitoring reference in veterinary medicine.

## 3. Methods

A comprehensive literature search was conducted in major databases, including Web of Science, Google Scholar, ScienceDirect, and PubMed. The search was performed using a set of predefined keyword combinations, with the primary focus on terms related to dog lung compliance. These included “canine lung compliance” and “canine respiratory compliance”.

Additionally, the keywords were adapted to align with the specific requirements of each section of the review. For the section on disease background, terms related to dog respiratory and cardiovascular disease were selected, including “obstructive ventilation disorders” and “restrictive ventilation disorders”, as well as other specific diseases, such as pneumonia, pulmonary edema, asthma, and so on. For the section on medicine, the keywords were related to drug classes, such as “anesthetics”, “analgesics”, “bronchodilators”, and so on, sometimes with the word “side effects”. For the section on surgery, the following keywords were used: “anesthesia”, “thoracoscopic surgery”, and “laparoscopic surgery”. In the initial stages of the writing process, 250 scientific works present in the literature were selected, encompassing a diverse range of formats, including case reports, reviews, research papers, and articles designed for a scientific audience. Subsequently, a literature screening was conducted to eliminate papers with a low relevance or those published too early in the field. Research papers, reviews, and clinical reports published after 2000 were selected for the analysis, except for some basic theoretical studies. In addition, some papers on human medicine were also retained for comparative reference. A total of 114 references were finally selected for this paper.

## 4. Breed and Size

In contrast to humans, dogs exhibit significant variations in body size, contingent on breed. These variations in size, proportions, and genetic characteristics influence thoracic volume, thereby directly contributing to disparities in the capacity of the lungs to expand adequately during inspiration [16,17,18,19]. A uniform and common unit of measurement (ml/cm H_2_O) is employed in the study of dog respiratory compliance, despite the existence of differences due to breed. Several studies have previously been conducted on this subject, yielding a variety of values for respiratory compliance, with these values differing depending on the breed of dog (Table 2).

Furthermore, Choi and Bradbrook in two separate studies proposed that dogs of varying sizes have different lung expansion capacities and lung tissue densities. Larger dogs exhibit a greater thoracic volume and a lower lung tissue density, enabling them to expand their lungs with greater ease, which will lead to a higher respiratory compliance [20,21].

Additionally, body size factors influence respiratory rate (RR) and tidal volume (VT). Large breeds have a lower respiratory rate but a larger tidal volume, while small breeds may have a higher respiratory rate but a smaller tidal volume. This also lends support to the idea that differences in body size can lead to differences in respiratory compliance. The results of a clinical study conducted in 2008, in which 78 dogs were evaluated for body weight and respiratory compliance, also demonstrated that dogs with higher body weights exhibited greater respiratory compliance [14]. In contrast, research has demonstrated that neither sex nor age has an impact on respiratory compliance in dogs [14,16].

**Table 2:** Reference range for dog respiratory compliance.

	Mean Weight (kg)	Weight Range (kg)	Respiratory Compliance Range (ml/cm H_2_O)	Mean Respiratory-Compliance-to-Bodyweight Ratio (C_T_/BW)	Measurement	Reference
Non-specific breeds		1.6–50.0	25.38 ± 20 *		Ventilator (Calculated via P-V loops)	[16]
		11.8–26.4	117			[19]
	26.8	1.9–45.0	32.831 *			[21]
	19.8			2.22		[18]
Specific Breeds						
Border collie	19.77		83.3 ± 5	3.31	Ventilator (Calculated via P-V loops)	[18]
German shepherd	31.0		121.0 ± 14.4	2.94		
Labrador retriever	27.5		77.6 ± 3.2	1.93		
Rottweiler	42.0		81.3 ± 7.3	1.33		

* These data are calculated from formulae given in the literature.

There is also evidence in human medicine that changes in body shape caused by disease, such as obesity, can have an impact on respiratory status. It has been suggested that obesity may lead to a decrease in thoracic volume, and that respiratory compliance is altered by external factors to a greater extent than in normal-sized patients [22,23]. Similarly, a 2020 clinical retrospective study observed that obese healthy dogs exhibited reduced respiratory compliance [17]. However, the study excluded dogs with respiratory disease and muscle relaxants, thereby preventing the determination of whether obesity exacerbates or directly contributes to alterations in respiratory compliance in dogs with pre-existing background diseases.

## 5. Recumbency

A 1992 study examined how different body positions (sternal, lateral, and supine) affect respiratory compliance in dogs. The results showed that lung volume distribution and ventilatory effects varied significantly with position due to gravity influencing regional lung pressure and alveolar expansion [22]. Furthermore, the effects of gravity and external compression result in different pulmonary perfusion volumes and thoracic volumes in canines in different positions, and this is thought to affect alveolar expansion and pulmonary compliance [23,24,25,26].

## 6. Diaphragm Movement

In a 2020 study, Yilmaz et al. studied 140 dogs that underwent computed tomography (CT), concluding that posture can affect dogs’ respiratory movements (particularly diaphragm movements) (Table 3) [24]. Moreover, prior research has also demonstrated that dogs undergoing anesthesia exhibit notable alterations in pulmonary perfusion volumes at various locations within their lungs, contingent on their body position [25]. Thus, in studies of respiratory compliance, this potential effect caused by the diaphragm should not be ignored.

A 1990 study observed that dog thoracic volume is dependent on the range of movement of the diaphragm [26]. A subsequent study conducted the following year also demonstrated that, regardless of posture, a longer initial diaphragmatic length allows the diaphragm to move farther, increasing thoracic volume and permitting a greater expansive movement of lung tissue [27].

However, the extant literature does not provide sufficient evidence to demonstrate that differences in thoracic volume due to diaphragmatic movement can directly determine differences in lung compliance.

## 7. Underlying Disorders

In the context of conducting studies on dog respiratory compliance, it is of paramount importance to screen for any underlying diseases that may be present. It is notable that dogs with different disease backgrounds exhibit markedly disparate changes in respiratory compliance (Table 4).

### 7.1. Restrictive Ventilation Disorder

Restrictive ventilation disorder is a condition in which the elasticity of the lungs or chest wall is reduced, usually in the form of a decrease in lung volume [28]. Reduced lung capacity means that lung tissue is unable to expand sufficiently, leading to a decrease in respiratory compliance.

**Figure 1:**
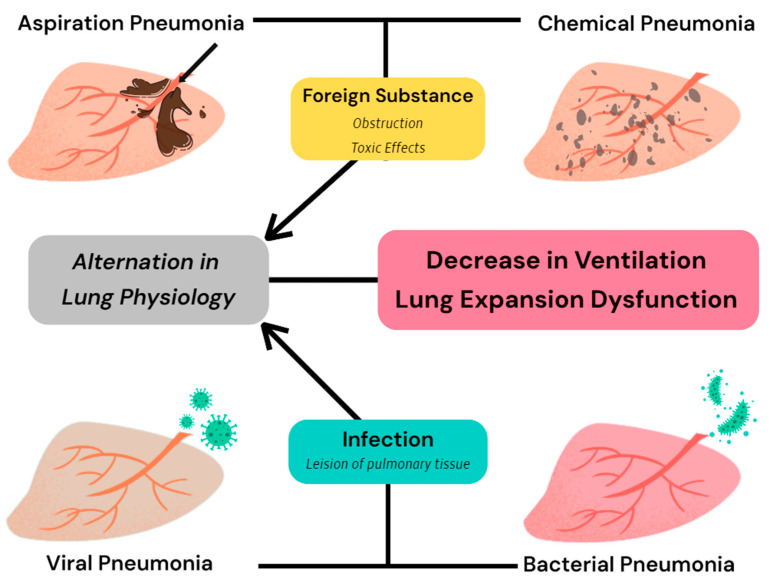
Four common types of pneumonia are all capable of inducing a reduction in the expansion capacity of lung tissue and alterations in the ventilatory status of dogs, although they do not exhibit the same pathological changes [29,30,31,32,33,34].

Pneumonia is a common disease of restrictive ventilatory failure, and although there are many causes of pneumonia, such as lung infections and foreign bodies (Figure 1) [29,30,31,32,33,34], a decreased expansion capacity of the lungs due to inflammatory lesions is the primary cause of restrictive ventilatory failure. A previous study on pneumonia and respiratory compliance in beagles in 1991 suggested that lower respiratory tract inflammation or pneumonia caused by viral infection may inhibit normal respiratory compliance in beagles during growth [35].

Pulmonary edema is also one of the common clinical conditions that can lead to restrictive ventilatory disorders in dogs. In 1985, Snapper et al. noted that pulmonary oedema reduces respiratory compliance: fluid accumulation in the interstitium is thought to significantly reduce intrapulmonary pressure, leading to the impairment of lung ventilation and normal lung tissue expansion [36]. In addition, pulmonary edema itself and the associated inflammation are thought to lead to a reduction in lung surfactant levels, further inhibiting normal tissue expansion and thus reducing respiratory compliance [37,38].

Unlike pulmonary edema, which acts within the interstitium of the lung to prevent lung tissue from expanding properly, pleural effusion (abnormal fluid in the chest cavity) disrupts the negative pressure environment within the chest cavity, reducing the volume of lung tissue that can expand. In a study published in 1993, researchers simulated the occurrence of pleural effusion by injecting saline into the chest cavity of dogs. They observed a significant reduction in the degree of elastic deformation of the chest cavity and airways in the experimental dogs, suggesting that dogs with pleural effusion are unable to expand the chest cavity as much as normal dogs, resulting in lung tissue not having enough volume to expand, leading to a reduction in respiratory compliance [39].

In addition, thoracic anomalies due to congenital dysplasia are not uncommon restrictive ventilatory disorders. Abnormalities of the vertebrae and sternum, including conditions such as funnel chest (PE) and pectus carinatum (PC), are often seen as manifestations of thoracic dysplasia [40,41]. Congenital thoracic malformations prevent the expansion of lung tissue during breathing in dogs, resulting in reduced respiratory compliance. This thoracic abnormality is more common in brachycephalic dogs and may exacerbate the decrease in obstructive ventilation caused by brachycephalic airway syndrome (BAS) [42].

### 7.2. Obstructive Ventilation Disorder

Obstructive ventilation disorder is a condition characterized by airway obstruction, increased breathing resistance, and significant dyspnea [28]. Reduced airway diameter and increased resistance can limit adequate alveolar expansion, resulting in reduced lung volume and further decreasing respiratory compliance [43]. This disorder encompasses various conditions, with the common cause being airway collapse or narrowing. Commonly, airway collapse is more prevalent in specific small breeds like Pomeranians, poodles, or obese dogs, where it can cause severe consequences [44,45,46,47].

Chronic obstructive pulmonary disease (COPD) and asthma are both classic obstructive ventilation disorders presenting persistent airway obstruction that progressively worsens over time. They are extremely dangerous for dogs, as well as humans [28,48]. Unfortunately, there is a paucity of direct studies of COPD in dogs. In comparative medicine studies utilizing animal models of rats and dogs, it has been observed that external factor stimuli (e.g., tobacco, dust, excessively dry air), specific immune responses, and even breed factors may contribute to the onset or progression of this type of disease [48,49,50,51,52,53]. In 2012, Papandrinopoulo et al. demonstrated in a study of human medicine that patients with COPD frequently exhibit decreased respiratory compliance due to the over-expansion of lung tissue from hyperinflation [54]. However, within the specific context of veterinary clinics, this aspect is understudied.

**Table 4:** Effects of different diseases on respiratory compliance in dogs.

	Mechanism	Effect on Respiratory Compliance	Reference
Restrictive ventilation disorder			
Pneumonia	Lung expansion inhibition Chest volume restriction Surfactant reduction	Reduce	[33,34,35]
Pulmonary edema			[36,37,38]
Pleural effusion			[39]
Thoracic anomalies			[40,41,42]
Obstructive ventilation disorder			
Tracheal collapse	Reduced tracheal diameter Serious bronchi obstruction Decreased ventilation Increased airway resistance	Reduce	[43,44,45,46,47]
COPD *			[48,49,50]
Emphysema			[51,52]
Asthma			[54]
BAS **			[55,56,57,58]
Abdominal disease			
Abdominal organs ectopic	Chest volume restriction Restricted movement of the diaphragm	Possibly reduce	[59]
Ascites			[60,61]

* COPD: chronic obstructive pulmonary disease; ** BAS: brachycephalic airway syndrome.

Brachycephalic airway syndrome (BAS) is one of the most common obstructive airway disorders in veterinary clinical practice today. The narrow nasal passages and elongated soft palate of BAS dogs result in reduced ventilation, directly contributing to decreased respiratory compliance [2,3,42,55,56,57]. Moreover, recent studies have also indicated that BAS dogs are more likely to have unanticipated airway obstruction during anesthesia, leading to the direct observation of reduced respiratory compliance [58].

### 7.3. Abdominal Disease

Although respiratory compliance is a measure of the degree of expansion of the lung tissue and the movement of the chest wall, it is well established within veterinary medicine, particularly in equine clinical practice, that the thoracic cavity may undergo a change in physiological state because of compression caused by ectopic or diseased abdominal organs. This results in a reduction in thoracic cavity volume, which in turn reduces the volume of lung tissue that can expand [59].

Abdominal diseases, such as ascites, have been demonstrated to result in diaphragmatic dyskinesia and respiratory depression. The severity of these conditions is directly proportional to the extent of ascites, with more pronounced diaphragmatic dyskinesia and pulmonary expansion dysfunction resulting in reduced thoracic volume [26,27,60]. Furthermore, traumatic diaphragmatic hernia is regarded as a prevalent condition in dogs. Dogs with such hernia always have cyanosis, and have abnormal breath rates in clinical practice. Moreover, hemothorax is also regarded as a complication of this disease, with the potential to result in alterations to the intrathoracic environment, thereby affecting the ventilatory status [61].

### 7.4. Obesity

There is also evidence in human medicine that changes in body shape caused by disease, such as obesity, can have an impact on respiratory status. It has been suggested that obesity may lead to a decrease in thoracic volume, and that respiratory compliance is altered by external factors to a greater extent than in normal-sized patients [62,63]. Similarly, a 2020 clinical retrospective study observed that obese healthy dogs exhibited reduced respiratory compliance [17]. However, the study excluded dogs with respiratory disease and muscle relaxants, thereby preventing the determination of whether obesity exacerbates or directly contributes to alterations in respiratory compliance in dogs with underlying disorders.

## 8. Pharmacological Influences

Obviously, drugs are one of the most crucial factors influencing the expression of respiratory compliance in animals (Table 5).

**Figure 2:**
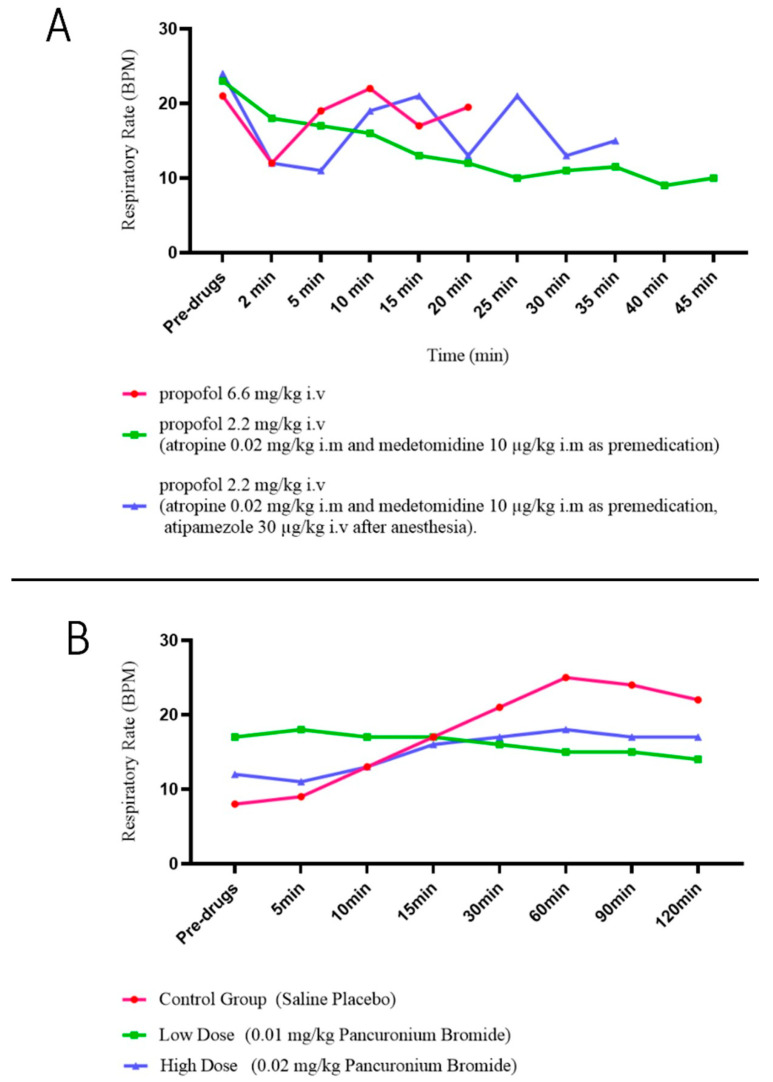
Effects of common drugs for surgery on respiratory rate in dogs: (**A**) propofol [66]; (**B**) pancuronium bromide (nondepolarizing muscle relaxant) [77]. These drugs can decrease the respiratory rate, leading to a possible decrease in respiratory compliance during surgery. These figures are modified from the stated references.

In a 2015 study, Saraswat pointed out that anesthetic drugs directly affect lung function [64]. Moreover, as early as the 1990s, Muir and Lin et al. noted in separate studies that propofol, a drug still commonly used to induce anesthesia, was able to cause respiratory depression, which is variable and common in clinical practice (Figure 2) [65,66]. Human medicine has also identified that propofol can inhibit respiratory muscles during surgery, particularly thoracic surgery. This effect, known as respiratory muscle relaxation, has been observed to worsen with increasing doses of the drug, the speed of injection, and the duration of surgery [67].

In addition, anesthetic gases currently used in veterinary medicine for maintenance anesthesia, such as desflurane, isoflurane, and sevoflurane, should also be considered. In 2001, a Japanese research team showed that currently used anesthetic gases did not induce changes in canine respiratory dynamics (e.g., dynamic respiratory compliance) at 1 MAC [68]. However, in 2003, a human study showed that anesthetic gases at 1 MAC decreased airway resistance and increased airway compliance, but desflurane at 2 MAC increased airway resistance and decreased airway compliance [69].

Analgesics and muscle relaxants are also commonly used in animal surgery. Although it has been suggested that both types of drugs may cause a dose-dependent respiratory depression and lead to a decrease in ventilation [70,71,72,73,74,75,76,77,78,79] (Figure 2) (Table 6), there are no studies suggesting that these drugs directly cause a decrease in respiratory compliance, although they have side effects like restrictive ventilatory disorders.

Salbutamol, a bronchodilator used in clinical practice, results in a substantial augmentation of minute ventilation (MV) in dogs, both in conscious and anesthetized states (Table 7) [80,81,82]. It has been proven that other drugs, like terbutaline and aminophylline, are efficacious in the dilation of the airway [83,84,85]. Aminophylline has also been shown to increase ventilation in awakening dogs and to have a positive inotropic effect on the diaphragm [84,85,86]. In human medicine, aminophylline has also demonstrated efficacy in improving respiratory muscle function after prolonged mechanical ventilation [87].

In addition to these classes of drugs, antithrombotic and antitumor drugs are increasingly used in veterinary medicine. However, respiratory side effects such as respiratory depression and dyspnea, and lung tissue lesions such as pulmonary edema and emphysema have been reported [88,89,90]. In addition, a 2008 clinical report documented the development of lung histopathology, including interstitial fibrosis and alveolar parenchymalisation, in a dog receiving lomustine (cumulative dose of 720 mg) for the treatment of lymphoma following the administration of the drug. The investigators suggested that such changes in lung tissue were related to antitumor effects [91], but did not address whether this would directly lead to changes in respiratory compliance.

**Table 5:** Overview of medications and their effects on ventilation and respiratory compliance in dogs, including mechanisms and potential impact.

	Specific Medicines	Mechanism	Potential Effect on Respiratory Compliance	Reference
Anesthetics	Propofol	Respiratory inhibition	Reduce	[65,66]
	Desflurane	Reduce respiratory resistance	Enhance or reduce (Dependent on MAC)	[68]
Analgesics	Morphine	Respiratory inhibition		[70,71,72]
	Sufentanil			[74]
	Remifentanil			[75]
Muscle relaxants	Pancuronium	Respiratory muscle depression		[77,78]
Bronchodilators	Salbutamol	Dilatation of trachea Increase ventilation Inotropic effects on diaphragms		[80,81,82]
	Aminophylline			[84,85,86]
Antithrombotics	Rivaroxaban	Respiratory depression		[88]
Antineoplastics	Doxorubicin	Side effects (pulmonary edema, interstitial fibrosis)		[89]
	Bleomycin			[90]
	Lomustine			[91]

**Table 6:** Respiratory inhibition caused by different doses of morphine in dogs [70,71].

Respiratory Inhibition Caused by Different Doses of Morphine in Dogs						
	Preinj Control *	5 min	10 min	30 min	60 min	120 min
End Tidal CO_2_ (mm Hg)						
0.9% NaCl (Control)	35.17			25.83	35.83	35.33
0.25 mg/kg Morphine	34.83			26.83	29.00	30.67
0.5 mg/kg Morphine	36.33			18.83	18.83	22.83
1.0 mg/kg Morphine	36.33			16.67	14.83	22.50
Tidal Volume (mL/kg)						
0.9% NaCl (Control)	11.33			12.31	11.78	10.81
0.25 mg/kg Morphine	12.52			9.52	9.00	8.47
0.5 mg/kg Morphine	12.32			6.92	6.35	6.64
1.0 mg/kg Morphine	11.15			5.21	4.55	5.99
Respiratory Rate (breaths/min, BPM)						
L. K.Cullen, M. R. Raffe et al. [70]						
0.9% NaCl (Control)	19.84			17.69	19.07	18.68
0.25 mg/kg Morphine	18.34			42.34	43.31	35.27
0.5 mg/kg Morphine	19.59			91.54	99.56	60.51
1.0 mg/kg Morphine	18.99			116.36	134.81	69.32
Kamata M., Nagahama S. et al. [71]						
0.3 × 10^−3^ mg/kg Morphine	30 ± 3	30 ± 6	31 ± 9	31 ± 3	32 ± 3	30 ± 4
0.6 × 10^−3^ mg/kg Morphine	37 ± 7	28 ± 3	32 ± 10	25 ± 3	31 ± 6	30 ± 2
1.2 × 10^−3^ mg/kg Morphine	34 ± 5	26 ± 7	24 ± 3	26 ± 3	23 ± 5	27 ± 5
2.4 × 10^−3^ mg/kg Morphine	30 ± 5	26 ± 2	25 ± 2	20 ± 4	22 ± 3	26 ± 2
Peak Expiratory Flow (L/min)						
0.9% NaCl (Control)	18.31			15.84	14.94	17.86
0.25 mg/kg Morphine	17.81			21.80	18.85	16.98
0.5 mg/kg Morphine	17.77			30.51	30.28	20.81
1.0 mg/kg Morphine	16.53			28.93	31.25	19.64

* Preinj Control: pre-injection control.

**Table 7:** Dogs can induce an increase in respiratory rate and tidal volume following salbutamol injection, which results in enhanced ventilation [81].

Ventilatory Response to Salbutamol in Dogs		
	Control	Salbutamol
Minute Ventilation (L/min)	10	16
Minute Volume per kg Body Weight (L/kg/min)		
Tidal Ventilation (mL)	370	480
Tidal Volume per kg Body Weight (mL/kg)		
Respiratory Rate (BPM)	27.6	34.9
Inspiratory Time (s)	1.06	0.94

## 9. Surgical Procedures

**Figure 3:**
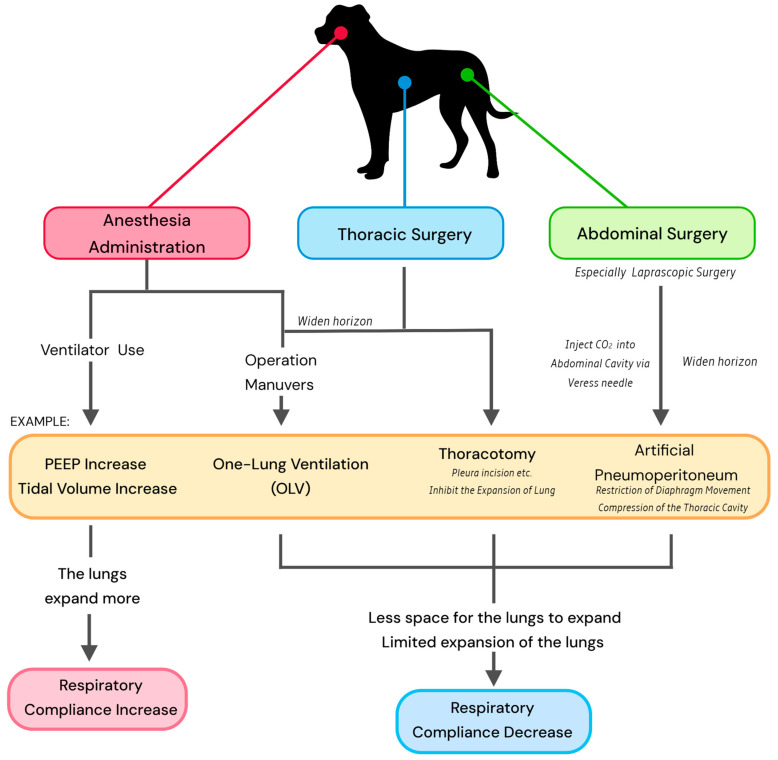
The effects of anesthesia management, operation maneuvers, and surgery type on respiratory compliance in dogs; in this figure, examples of each factor are given in the yellow bubble to demonstrate the simple mechanism.

Surgery and related maneuvers affect respiratory compliance due to the alteration in ventilation status in surgery (Figure 3).

### 9.1. Anesthesia

It is currently believed that respiratory compliance in dogs can be accurately monitored by ventilators under anesthesia [11,16]. Consequently, in most clinical cases, respiratory compliance is often assessed at the time of surgery, allowing any complications from the procedure to be considered. During this phase, the veterinarian can regulate and monitor the animal’s respiratory status and parameters, including ventilation, tidal volume, and airway pressure during respiration, via a ventilator. This allows the veterinarian to obtain respiratory information regarding the P-V loop, which provides valuable information about the animal’s ventilatory function [1,2,3,4,16,92]. This approach offers a more intuitive and accurate method of responding to an animal’s respiratory compliance compared to clinical lung function testing in animals [2,11,12,13,16].

Although tracheal intubation is not practiced in all veterinary clinical procedures, it is a mandatory procedure if accurate respiratory compliance is to be obtained. However, in 2024, Raillard et al. noted that since tracheal intubation is usually determined by the size of the dog, changes in respiratory mechanics due to different intubations are usually already included in the differences due to size. Thus, it is not necessary to include tracheal intubation as a separate factor in the main considerations [93].

As early as 1974, one study based on anesthetized rats showed that positive end-expiratory pressure (PEEP) adjusted by a ventilator was effective in reducing pulmonary oedema due to hyperinflation and improving lung compliance in anaesthetized animals [94]. And in recent years, more studies have focused on this aspect. A 2022 study on horses conducted by Andrade et al. demonstrated that appropriate PEEP was an effective intervention for enhancing respiratory compliance. This increase in respiratory compliance was associated with favorable lung tissue recovery, reduced lung damage, and improved arterial oxygenation [95]. In a separate study conducted in the same year, Araos et al. similarly observed that appropriate PEEP facilitates optimal lung expansion during dog surgical procedures [96]. In 2023, Zersen’s study also demonstrated that PEEP was an effective intervention for increasing respiratory compliance in dogs undergoing surgery. This increase in respiratory compliance was also considered to be an indication of good alveolar recruitment [2]. These studies conclude that respiratory compliance serves as a direct indicator of the extent of recruitment of lung tissue in response to a PEEP setup performed by a veterinarian. Additionally, it can be inferred that higher respiratory compliance is indicative of reduced lung tissue damage and enhanced capacity for lung expansion, which in turn facilitates improved respiratory motion and oxygenation.

### 9.2. Thoracic Surgery

Thoracic surgery has been proposed as a potential contributor to altered respiratory compliance, given the possibility of trauma resulting from surgical procedures, which could subsequently lead to damage to lung tissue. A 2007 study in human medicine also noted that reduced respiratory compliance due to pharmacological factors can be more pronounced in thoracic surgery than in other procedures [67].

Research from 2015 indicates that the trauma caused by veterinary surgery is comparable to that caused by human surgery [97]. A considerable proportion of patients (nearly 50%) have been observed to present with a range of lung tissue damage, with similar clinical manifestations, including lung collapse and ischemic reperfusion injury [98]. In dogs, the incidence of post-operative and intra-operative complications from thoracic surgery is almost 90% (83 dogs and cats in total in the study) [99]. Respiratory compliance can be significantly reduced by any condition that interferes with the normal expansion of the lungs.

In the context of advancements in veterinary surgery, there has been a notable increase in the utilization of one-lung ventilation (OLV) techniques in thoracic surgery, particularly in the domain of video-assisted thoracic surgery (VATS). OLV is a technique used in thoracic surgeries where one lung is ventilated while the other is collapsed, or not ventilated artificially, to improve the surgeon’s access to the chest cavity and minimize interference from the ventilated lung [9,99,100]. However, this maneuver can also lead to a reduction in pulmonary compliance or respiratory compliance as the lung tissue cannot reach sufficient expansion, in severe cases leading to more serious postoperative complications, such as artificial pneumothorax [98]. The utilization of LPV is advocated in human medicine to avert potential lung tissue damage arising from OLV. It has been hypothesized that compliance can serve as a monitor of lung ventilation during OLV [101]. However, this recommendation has not been popularized in veterinary clinics.

Furthermore, dogs diagnosed with congenital heart disease are advised to undergo surgical intervention, including patent ductus arteriosus (PDA) [102]. Due to cardiac disease, with a particular emphasis on left heart disease, there is an increased propensity for respiratory complications, such as cardiogenic pulmonary edema, to arise [103,104,105]. The potential anesthetic risks associated with cardiac disease are not negligible even in minimally invasive procedures such as thoracoscopy [9]. It is therefore recommended that veterinary surgeons should treat dogs with cardiac disease as potentially more susceptible to altered respiratory compliance and monitor them more carefully under anesthesia when performing thoracic surgery, despite the current lack of research evidence to support this approach.

### 9.3. Abdominal Surgery

A 2020 study concluded that, in healthy dogs, the type of surgery has minimal impact on respiratory compliance, while anesthesia and recumbency are dominant factors [17]. Abdominal surgery inevitably requires dogs to remain in the supine position for an extended period. In this position, lung expansion is partially restricted by gravity, and diaphragmatic movement is limited, reducing thoracic cavity volume and, consequently, respiratory compliance [22,23,24,25,26].

Furthermore, respiratory compliance constitutes a significant component of the anesthetic evaluation in dogs undergoing laparoscopic surgery. Most laparoscopic surgeries involve the creation of pneumoperitoneum by insufflating gas into the abdomen to facilitate minimally invasive techniques [106]. Intra-abdominal pressure (IAP) reflects the amount of gas used for an artificial pneumoperitoneum. Excessive IAP has been shown to restrict diaphragmatic movement and reduce respiratory compliance in veterinary studies [58,106,107,108,109]. Additionally, carbon dioxide (CO_2_) is commonly used for insufflation, which can elevate end-tidal CO_2_ (ETCO_2_) levels due to its absorption into the splanchnic circulation, potentially reducing respiratory compliance [110].

## 10. Current Applications and Prospects

Recent studies have demonstrated that respiratory compliance can serve as an indicator of the extent of lung recruitment during surgical procedures in small animals. This finding suggests that veterinarians can assess and adjust PEEP settings based on respiratory compliance values measured and provided by the ventilator to enhance ventilation in the animal [2,93,94,95]. As in human medicine, it is currently recommended that veterinary anesthetists perform ARMs during surgical procedures, particularly laparoscopic surgery, to prevent lung tissue damage, such as lung collapse, caused by excessive IAP during surgery [109]. Despite evidence from earlier studies that alveolar recruitment can result in lung tissue injury due to hyperinflation, this can be circumvented. Some studies have demonstrated that the protective effect on lung tissue from either mode of ARM (sustained inflation ARM or stepwise ARM) significantly outweighs any potential adverse effects. Furthermore, ARMs can be effectively controlled by the anesthetist through the monitoring of respiratory compliance [10,109,110,111]. In the field of human medicine, ARMs have been demonstrated to be more beneficial to the postoperative respiratory status of patients undergoing cardiac surgery and laparoscopic surgery, which can help to improve pulmonary compliance, especially at higher IAP [112,113].

Ventilators used in veterinary clinical practice are now almost equipped with the capability to accurately measure and regulate system pressure and volume, which assists veterinarians in establishing a safer and more stable anesthetic respiratory state during surgery. However, the cost and lack of demand have resulted in not all ventilators being equipped with a module specifically designed to measure respiratory compliance. Consequently, veterinary anesthetists lack a suitable standard (e.g., the C_dyn_ standard for dogs) to assess whether they are achieving the desired results or causing unintended ventilator-induced lung injury when using PEEP for ARMs. Furthermore, C_st_ can be measured by temporary artificial respiratory pauses to estimate airway resistance during surgery. However, in practice, there is a lack of willingness among veterinarians to stop the respiration of surgical animals except when deemed essential (e.g., intraoperative C-arm angiography).

Furthermore, C_st_ has been found to be overly sensitive to acute respiratory failure syndrome (ARDS) in human medical practice, especially in pediatric patients [114]. Analyzing these data in conjunction with arterial blood gases is a well-established method of confirming the diagnosis of this disease. However, this is difficult to perform within the veterinary field due to the poor cooperation of the animal. The accuracy of measuring respiratory compliance for conscious dogs by spirometry is poor [11,13]. Consequently, it remains challenging to capture respiratory compliance while the animal is awake and use this as a measure for clinical diagnosis.

## 11. Conclusions

The use of respiratory compliance, whether static (C_st_) or dynamic (C_dyn_), remains limited in clinical studies and applications, primarily due to the constraints of monitoring instruments.

While respiratory compliance monitoring is used in veterinary surgery, particularly in thoracic and laparoscopic procedures, it is not yet universally adopted. Not all veterinarians—or more precisely, anesthetists—choose to assess ventilation status or alveolar recruitment maneuvers (ARMs) using respiratory compliance during surgery. We argue that to minimize anesthetic risk, animal hospitals equipped with respiratory compliance monitoring should incorporate it as a key parameter in anesthetic monitoring, especially during thoracic and laparoscopic surgeries.

Furthermore, the practical challenges of monitoring respiratory compliance in awake animals are well recognized, despite the recognized value of respiratory compliance in the diagnosis of respiratory diseases in human medicine. However, we propose that measuring respiratory compliance in awake animals is a promising area for future research and that veterinary clinics should explore methods and feasibility.

## Figures and Tables

**Table 1:** Differences between static and dynamic respiratory compliance [1,2,3,4,5].

	Static Compliance (C_st_)	Dynamic Compliance (C_dyn_)
Calculation Method	$C_{st}=\frac{V_{t}}{P_{plat}-PEEP}$	$C_{dyn}=\frac{V_{t}}{PIP-PEEP}$
Unit of Measurement	Milliliters per centimeter of water column (ml/cm H_2_O) Liters per centimeter of water column (L/cm H_2_O)	
Measurement Conditions	Ventilation pauses under anesthesia	Mechanical ventilation under anesthesia.
Reflects	Elasticity of the lungs Compliance of lung tissue	Ventilation states
Main Influencing Factors	Lung tissue elasticity Thoracic compliance Respiratory muscle function	Airway resistance Mechanical properties of the airway Ventilator settings and ventilation modalities

C_st_: static compliance; C_dyn_: dynamic compliance; V_t_: tidal volume; P_plat_: plateau pressure; PIP: peak inspiratory pressure; PEEP: positive end-expiratory pressure.

**Table 3:** Differences in tissue and air volume in the lungs due to different body positions of dogs [26].

Differences in Tissue and Air Volume in the Lungs Due to Different Body Positions of Dogs		
	Supine	Prone
Air Volume (mL/kg)		
L cranial	13.7 ± 4.1	13.3 ± 3.4
L middle	8.8 ± 3.0	8.6 ± 2.9
L caudal	27.6 ± 5.1	27.9 ± 4.5
L lung	50.1 ± 11.9	49.8 ± 10.5
Whole lung	118.7 ± 26.4	118 ± 24.5
Tissue Volume (mL/kg)		
L cranial	1.17 ± 0.30	1.35 ± 0.34
L middle	0.74 ± 0.17	0.93 ± 0.29
L caudal	2.37 ± 0.43	2.60 ± 0.53
L lung	4.28 ± 0.81	4.88 ± 1.08
Whole lung	10.16 ± 1.83	11.57 ± 2.25

## Data Availability

No new data were created or analyzed in this study.
